# Self-Assembly M2e-Based Peptide Nanovaccine Confers Broad Protection Against Influenza Viruses

**DOI:** 10.3389/fmicb.2020.01961

**Published:** 2020-08-14

**Authors:** Qimin Wang, Yuling Zhang, Peng Zou, Meixiang Wang, Weihui Fu, Jialei She, Zhigang Song, Jianqing Xu, Jinghe Huang, Fan Wu

**Affiliations:** ^1^Shanghai Public Health Clinical Center, Fudan University, Shanghai, China; ^2^Key Laboratory of Medical Molecular Virology (MOE/NHC/CAMS), School of Basic Medical Sciences and Shanghai Public Health Clinical Center, Fudan University, Shanghai, China

**Keywords:** influenza, M2e, peptide, self-assembly, nanoparticle

## Abstract

The extracellular domain of influenza M2 protein (M2e) is highly conserved and is a promising target for development of universal influenza vaccines. Here, we synthesized a peptide vaccine consisting of M2e epitope linked to a fibrillizing peptide, which could self-assemble into nanoparticle in physiological salt solutions. When administrated into mice without additional adjuvant, the influenza A M2e epitope-bearing nanoparticles induced antibodies against M2e of different influenza subtypes. Comparing with other M2e-based vaccine, these M2e nanoparticles did not induce immune response against the fibrillizing peptide, demonstrating minimal immunogenicity of vaccine carrier. Furthermore, vaccination with M2e-based nanoparticles did not only protect mice against homologous challenge of influenza PR8 H1N1 virus, but also provide protection against heterologous challenge of highly pathogenic avian influenza H7N9 virus. These results indicated that M2e-based self-assembled nanoparticle vaccine is safe and can elicit cross-protection, therefore is a promising candidate of universal influenza vaccines.

## Introduction

Influenza remains as a great threat to public health despite that it has been studied for over 100 years since 1918. The seasonal flu epidemics cause about 3–5 million cases of severe illness and 290,000 to 650,000 deaths around the world annually (World Health Organization [WHO], 2018). Current seasonal influenza vaccines, including inactivated and live-attenuated influenza vaccines, provide protection by inducing neutralizing antibodies against the influenza hemagglutinin protein (HA) (Caton et al., 1982). Viruses could escape from the protection by rapid antigenic drift due to the accumulation of mutations on the antibody binding site of HA protein and make vaccine ineffective (Ilyushina et al., 2019). The antigenic components of influenza vaccine need to be updated every year to catch up with the change of epidemic viruses (Krammer and Palese, 2015). The viral antigen components in seasonal vaccines are selected based on the influenza surveillance data and epidemic prediction (Auladell et al., 2019). Seasonal flu vaccine efficacy is determined by the similarity between the vaccine strains and circulating viruses (Estrada and Schultz-Cherry, 2019). The vaccine may not be able to provide enough protection when mismatched with the circulating strains (Belongia et al., 2016), resulting in the outbreak of influenza infection. It has been suggested that the ineffectiveness of seasonal flu vaccine caused significant morbidity and mortality worldwide during the 2017–2018 flu season (Barr et al., 2018). Furthermore, the appearance of highly pathogenic avian influenza viruses, including H5N1 and H7N9 viruses, poses challenges on the current strategy of influenza vaccine by emerging novel influenza strains. Avian influenza viruses may cause worldwide influenza pandemic if they acquire efficient transmission between humans. Current seasonal influenza vaccine could not provide efficient protection against avian influenza viruses (Stephenson et al., 2004; Zhou et al., 2013). There is an urgent need to improve vaccine strategy against influenza and to develop universal influenza vaccines that could provide cross-protection against different influenza strains.

The ectodomain of influenza M2 protein (M2e) is a promising target for developing universal influenza vaccines (Estrada and Schultz-Cherry, 2019). M2e contains 23 amino acids and is highly conserved across multiple influenza strains. Passive administration of M2e-specific monoclonal antibodies was able to provide protection against influenza challenge in experimental animals (Zebedee and Lamb, 1988; Liu et al., 2004). A M2e-specific human monoclonal antibody could reduce clinical symptoms and viral loads when given to volunteers who were challenged with influenza epidemic strains (Ramos et al., 2015). M2e-elicited protective immunity has been achieved in animals by vaccination with different types of M2e-based influenza vaccines including M2e peptide conjugates, recombinant M2e fusion proteins, M2e-based DNA vaccines and others. Several M2e influenza vaccines have been evaluated in phase I/II clinical trials, yet there has not been M2e-based influenza vaccine available for clinical use (Kolpe et al., 2017).

Due to the poor immunogenicity of the pure M2e protein, most of M2e-based vaccines used carrier proteins, chemically or genetically fused with M2e, to enhance the immunogenicity of M2e vaccines (Estrada and Schultz-Cherry, 2019; Mezhenskaya et al., 2019). These vaccines did not only successfully provoke M2e-specific immune responses but also induced strong immune responses to the carrier proteins, which may cause unexpected side effect when used in humans. For example, an M2e-based influenza vaccine VAX102, which is a recombinant M2e–flagellin fusion protein, caused strong local and systemic adverse reactions when given in high dose to humans (Turley et al., 2011). Strong carrier-specific immune response may also cause carrier-induced epitopic suppression and so attenuate M2e-specific immune response (Heinen et al., 2002). Another M2e-based vaccine ACAM-FLU-A, which consists of three tandem copies of M2e epitope fused to hepatitis B core protein, induced M2e-specific antibodies in a phase I clinical trial (ClinicalTrials.gov Identifier No. NCT00819013). However, the titers of M2e-specific antibodies decreased rapidly over time in the volunteers (Kolpe et al., 2017). Although the detailed results and underlying mechanisms have not been published yet, we speculated that the decrease of M2e-specific antibodies may be due to the strong immunogenicity of HBV core protein since carrier-induced epitopic suppression was previously observed in epitope conjugate vaccines (Jegerlehner et al., 2010).

In this study, we performed proof-of-concept research and evaluated the safety and effectiveness of a novel method to enhance the immunogenicity of M2e epitope. We synthesized a peptide that consists of M2e epitope linked to a fibrillized peptide Q11. The Q11 peptide could self-assemble into a nanofiber in physiological pH condition and act as immune adjuvant to enhance the immunogenicity of the linked antigenic epitopes (Rudra et al., 2010, 2012a,b). We validated that the M2e-Q11 peptide could self-assemble into nanoparticles and provoke M2e-specific antibody response in mice without additional adjuvant. Furthermore, M2e-Q11 nanoparticles did not induce immune responses against the fibrillizing Q11 peptide, reducing the potential side effects that could be caused by the immunogenicity of the vaccine carrier. Vaccination with M2e-Q11 nanoparticles was also able to protect mice against lethal does challenge of different influenza subtypes, suggesting that the self-assembly of M2e-Q11 nanoparticles is a promising strategy for developing universal influenza vaccine.

## Materials and Methods

### Peptides and Virus

Peptides were synthesized by solid phase method in Synpeptide Co. (Shanghai, China). M2e peptide, N-SLLTEVETPIRN EWGCRCNDSSD, is a conserved M2e domain of human influenza A virus. Q11 peptide, N-QQKFQFQFEQQ, is a self- assembly peptide which forms into fibril in physiological pH conditions. M2e-Q11 peptide, N-SLLTEVETPIRNEW GCRCNDSSDSGSGQQKFQFQFEQQ, is the M2e peptide covalently linked to Q11 peptide with spacer sequence GSGS. Avian M2e peptide, N-SLLTEVETPTRTGWECNCSGSSD, is a peptide containing M2e sequence from avian H7N9 virus. Swine M2e peptide, N-SLLTEVETPTRSEWECRCSGSSD, is a peptide containing M2e sequence from swine H1N1 influenza virus. All the peptides were purified by HPLC and analyzed by mass spectrometry. The purity of the peptides was above 90%. Stock solution of the peptides was prepared at the concentration of 8 mM in distilled water and stored at −20°C.

Mouse-adapted influenza viruses, A/Purto Rico/8/34 (PR8, H1N1) and avian influenza virus A/Shanghai/4664T/2013 (H7N9) were propagated in MDCK cells. The TCID50 and plague forming units (PFU) of viruses were evaluated on MDCK cell monolayer. The lethal doses of viruses was titrated on mice and calculated by Reed–Muench method. The experiments involved avian H7N9 influenza virus were conducted in ABSL-3 lab in Shanghai public health center and under protocols approved by the institutional biosafety committee.

### Nanoparticle Size Determination

Peptides M2e-Q11, Q11, and M2e were diluted from the stocking concentration (8 mM) to 1mM with PBS and incubated at room temperature for 4 h to allow fibrilization. The solutions were further incubated overnight at 4°C before being diluted with PBS to a final concentration of 0.25 mM. Peptide solutions were adsorbed onto carbon-coated 200 mesh copper grids and negatively stained with 2% uranyl acetate and imaged with a FEI Tecnai G2 Spirit transmission electron Microscope. The size distributions of nanoparticles were analyzed by dynamic light scattering with Zetasizer ZEN3600 (Malvern Panalytical Ltd.).

### Immunization

Female Balb/C mice (6–8 weeks old) were divided into four groups and intraperitoneally immunized with 10 nM of M2e-Q11 peptide, Q11 peptide and M2e peptide, respectively. Prior to immunization, M2e-Q11and Q11 peptides were diluted into sterile PBS solution at the concentration of 2 mM and incubated at room temperature for fibrilization. M2e peptide was diluted into 200 μM and 1:1 mixed with aluminum adjuvant (InvivoGen 5200). Mice were intraperitoneally immunized for 10 nM nanoparticle or peptide each. Boosting immunization was given with the same vaccine formula 2 weeks post the first immunization. Sera were collected 14 days after the booster. Sera from mice immunized with PBS were collected as negative controls.

### Antibody Detection

M2e and Q11 specific antibodies were measured by Enzyme-linked Immunosorbent Assay (ELISA). Briefly, Nunc-immuno plates (Nunc 442404) were coated with 50 μL PBS-diluted human M2e peptide, avian M2e peptide, or swine M2e peptide, 5 μg/mL, at 4°C for overnight. Unspecific binding was blocked with 200 μL of 0.25% gelatin in PBST solution (0.5% Tween-20 in PBS). 50 μL of the serially diluted sera was added to each well and incubated at 37°C for one and half hours. After extensive washing with PBST solution, binding antibodies were detected by HRP labeled goat anti-mouse IgG antibody (BioLegend B243363) and TMB substrate (Biosharp 0759), accordingly to manufacturer’s instructions.

The isotypes of M2e-specific antibodies were determined by ELISA with Mouse Monoclonal Antibody Isotyping Reagents (Sigma, ISO-2) following the manufacturer’s instructions.

### Influenza Virus Challenge

Mice were anesthetized by isoflurane (Baxter CN2L9100) and intranasally challenged with 5 LD50 of mouse-adapted PR8 (5 × 10^3^ TCID_50_) and avian H7N9 (1.75 × 10^4^ TCID_50_) influenza viruses, 3 weeks post the final immunization. Mouse body weight and survival rates were monitored daily for 2 weeks post infection.

### Detection of Pulmonary Virus Titers by RT-qPCR

At the 14th day of challenge, the surviving mice were deeply anesthetized and decapitated, and their lung tissues were taken for RNA extraction (Tiangen DP431). Primers and probe targeting the M gene of influenza and the β-actin of mouse were used for RT-qPCR with the following sequences: 1012FluA-Fv1 GGARTGGMTAAAGACAAGAC CAATC; 1012FluA-Rv1 GGCRTTYTGGACAAASCGTCTAC; 1012FluA-Pv1 5′ ROX-AGTCCTCGCTCACTGGGCACGGT-3′ BHQ2; b-actin Forward GAGATTACTGCTCTGGCTCCTA; b-actin Reverse GGACTCATCGTACTCCTGCTTG; b-actin P 5′VIC- CCTGAGCGCAAGTACTCTGTGTGGATC-3′ BHQ. RT-qPCR was performed using the ABI 7500 detection system in One Step PrimeScript^TM^ RT-PCR Kit (Perfect Real Time) (Takara RR064A) with the following conditions: 42°C for 10 min, 95°C for 2 min, and 40 cycles of 95°C for 10 s and 60°C for 1 min. Threshold cycle (CT) values representing viral genomes were analyzed with CFX Manager software, and the data were shown as genome equivalents (GEq) per microliter.

### Statistical Analysis

Antibody titers, viral titers, and body weights between groups were compared by 1- or 2-way ANOVA. The survival rate was compared by Log-rank test. *P*-value of less than 0.05 was considered as significant. All the statistical analysis was performed by GraphPad Prism version 7.00 (GraphPad Software, San Diego, CA, United States).

## Results

### M2e-Q11 Peptide Assembled Into Nanostructure in PBS Solution

**FIGURE 1:**
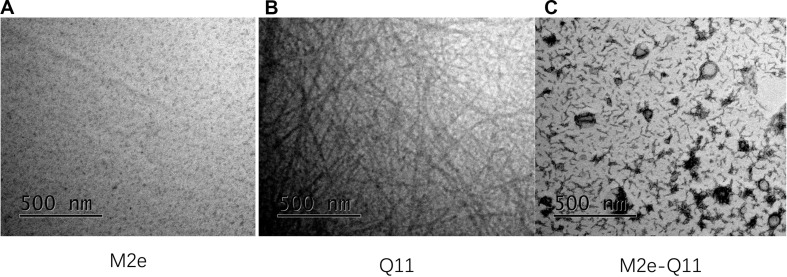
Nanostructure of self-assembly peptides by Transmission electron microscope (TEM). Peptides were diluted into PBS solution and incubated overnight to assemble into nanostructure. Peptide solutions were adsorbed onto carbon-coated copper grids and negatively stained with uranyl acetate and imaged with a FEI Tecnai G2 Spirit TEM. **(A)** M2e peptide; **(B)** Q11 peptide; **(C)** M2e-Q11 peptide.

**FIGURE 2:**
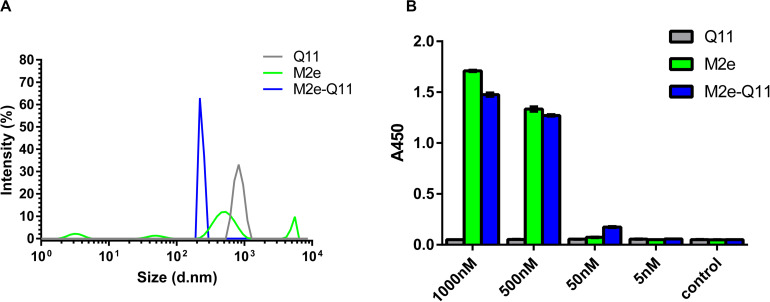
Characterization of M2e-Q11 nanoparticles. **(A)** Size distribution of Q11, M2e-Q11 peptide nanoparticles and M2e peptide. **(B)** Reactivity of protective anti-M2e mAb 8C6 to self-assembly nanoparticles. Plates were coated with peptide or peptide-formed nanoparticles at different concentrations. Binding of 8C6 to peptide or nanoparticles were measured by ELISA. Each sample was tested in triplicate and mean values of absorbance at 450 nm (A450) are shown.

Influenza M2e epitope consists of only 23 amino acids and is poorly immunogenic when administered alone. We previously reported that a 23-mer M2e peptide could partially self-assemble into polymer through intra-peptide disulfide bonds and induce M2e-specific immune response with adjuvants in experimental mice (Wu et al., 2007; Zou et al., 2017). In this study, we synthesized a 38-mer M2e-Q11 peptide, SLLT EVETPIRNEWGCRCNDSSDSGSGQQKFQFQFEQQ, consists of human influenza M2e epitope linked to a fibrillizing peptide Q11 domain (QQKFQFQFEQQ) with four amino acid Ser-Gly-Ser-Gly as spacer. The Q11 peptide was used to promote the peptide assembly into nanoparticle since it has been successfully used as immune adjuvant to enhance immunogenicity of ovalbumin and malaria epitopes. The M2e and Q11 peptides were also synthesized as controls. All the peptides dissolved in water at 10 mg/ml without precipitation. When diluted into PBS solution, M2e-Q11 and Q11 peptides assembled into different nanostructure, which were observed under TEM and shown in Figure 1. Q11 peptide self-assembled into fibrous structure as previously described (Rudra et al., 2010). However, M2e-Q11 peptide did not form long nanofibers as the Q11 peptide did, but assembled into branched nano sticks instead. The branched nano sticks were about 100 nm in length and 15 nm in width. Despite that the M2e peptide could form peptide polymer by intra-peptide disulfide interaction, it did not form detectable nanostructure under TEM. The size distributions of the nanoparticles were analyzed by dynamic light scattering analysis and shown in Figure 2A. The average hydrodynamic diameter was 776 ± 147 nm for Q11 peptide nanoparticles and 238 ± 25 nm for M2e-Q11 peptide nanoparticles.

Next, we used an M2e-specific monoclonal antibody 8C6 to evaluate whether M2e epitope was correctly presented on the M2e-Q11 nanoparticles. 8C6 was isolated from M2e-immunized mouse and could protect against lethal dose of influenza challenge when passively administered into mice (Liu et al., 2004). The interaction of mAb 8C6 with M2e-Q11, Q11 nanoparticles and M2e peptides were evaluated by ELISA. As shown in Figure 2, 8C6 efficiently recognized M2e-Q11 nanoparticles and M2e peptide, but was not able to bind the Q11 nanofibers. These results suggested that the protective M2e epitope was correctly presented on M2e-Q11 nanoparticles and accessible to protective antibodies.

### M2e-Q11 Nanoparticles Induced Antibodies Recognizing M2e Epitopes of Human, Swine, and Avian Influenza Viruses

**FIGURE 3:**
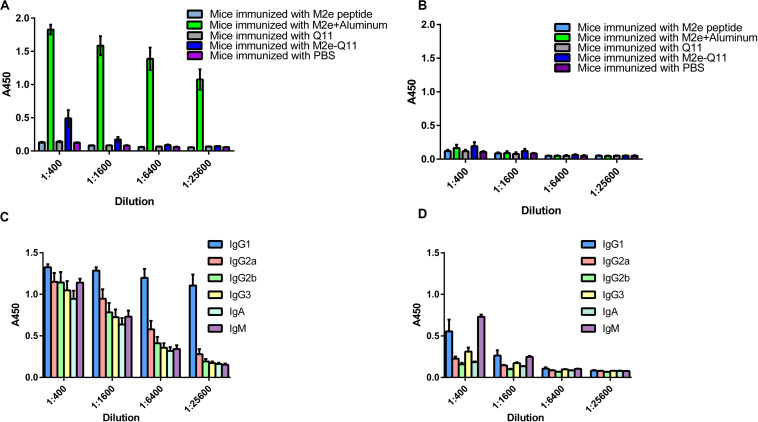
M2e-specific and Q11-secific antibodies were determined by ELISA. Mice were immunized with M2e-Q11 nanoparticle, aluminum-adjuvant M2e peptide, Q11 nanofiber, M2e peptide or PBS alone. Sera were collected 14 days after twice immunization. Antibodies specific for human influenza M2e peptide **(A)** or Q11 peptide **(B)** were tested by ELISA. Average values of absorbance at 450 nm (A450) of eight mice in each group are shown. The isotypes of M2e-specific antibodies in mice immunized with aluminum-adjuvant M2e peptide **(C)** and M2e-Q11 peptide nanoparticles **(D)** are shown.

We evaluated whether M2e-Q11 nanoparticles were able to induce M2e-specific immune response. Balb/c mice were intraperitoneally immunized with M2e-Q11 nanoparticles or Q11 nanofiber without adjuvants. Mice were also intraperitoneally immunized of M2e peptide with aluminum adjuvant as positive control. Mouse sera were collected 14 days after the boosting immunization. M2e-specific and Q11-specific antibodies were evaluated by ELISA. As shown in Figure 3A, M2e-specific antibodies were induced in mice immunized with M2e-Q11 nanoparticles. The titers of M2e-specific antibody in mice immunized with M2e-Q11 nanoparticles were about 1:1600, which were lower than those in mice immunized with aluminum-adjuvant-M2e peptide. As expected, M2e-specific antibodies were not detected in mice immunized with Q11 nanofiber or PBS solution. Consistent with previous reports, neither M2e-Q11 nanoparticles nor Q11 nanofiber was able to induce Q11-specific antibodies (Figure 3B). Comparing to aluminum-adjuvant-M2e peptide which induced multiple isotype of M2e-specific antibodies (Figure 3C), M2e-Q11 nanoparticles mainly induced IgG1 and IgM isotypes of M2e-specific antibodies (Figure 3D).

**FIGURE 4:**
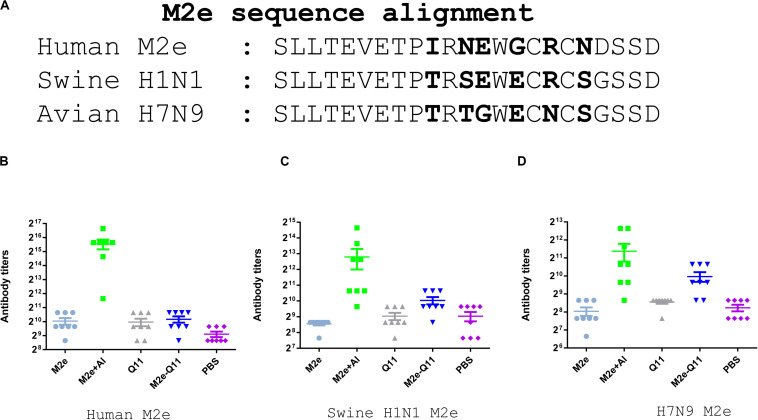
Evaluation of antibodies cross-reactive with swine H1N1 and Avian H7N9 M2e peptide. **(A)** Sequence alignment of human influenza, swine H1N1 and avian H7N9 M2e sequences. The amino acid substitutions were highlighted in bold. Antibodies titers specific for human M2e **(B)**, swine H1N1 influenza M2e **(C)** and avian H7N9 M2e **(D)** are shown.

We further evaluated whether antibodies induced by M2e-Q11 nanoparticles could cross-react with M2e proteins of other influenza subtypes. We synthesized M2e peptides according to M2e sequences from swine H1N1 and avian H7N9 influenza viruses and tested the cross-reactivity of antisera to these peptides by ELISA. There are six amino acid substitutions among human M2e, swine H1N1 and avian H7N9 M2e sequences (Figure 4A). As shown in Figures 4B–D, sera from M2e-Q11 vaccinated mice efficiently bind to M2e peptides from avian H7N9 and swine H1N1 influenza virus despite that there were six amino acid substitutions in human M2e versus swine and avian M2e sequences. The titers of antibodies from M2e-Q11 immunized mice were about 1024 against avian and swine M2e, which were similar to the titers against human influenza M2e. We observed that aluminum-adjuvant-M2e peptide vaccine also induced antibodies against avian and swine M2e peptide but antibody titers against swine and avian influenza M2e peptides were about fourfold lower than that against human M2e peptide.

### Vaccinated With M2e-Q11 Nanoparticle Protected Mice Against H1N1 and Avian H7N9 Influenza Challenge

**FIGURE 5:**
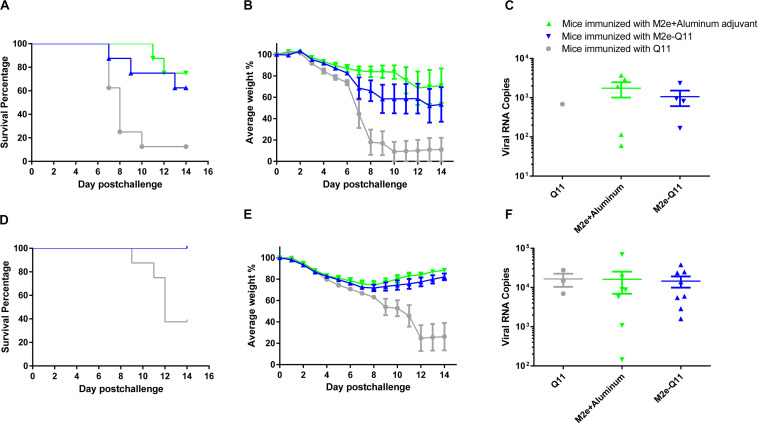
Vaccination with M2e-Q11 nanoparticle conferred protection against PR8 and avian H7N9 influenza challenge. **(A)** Mice were immunized with M2e-Q11 nanoparticle, aluminum-adjuvant M2e peptide or Q11 nanofiber and challenged with 5 LD_50_ (5 × 10^3^ TCID50) of mouse-adapted influenza PR8 virus (A/Purto Rico/8/34, H1N1). The survival curves were analyzed by Kaplan–Meier methods (*N* = 8 in each group). **(B)** Average weight of eight mice challenged with influenza PR8 virus in each group are shown. **(C)** Viral RNA copies in the lung tissues of survivors in each group challenged with influenza PR8 virus are shown. **(D)** Mice were immunized with M2e-Q11 nanoparticle, aluminum-adjuvant M2e peptide or Q11 nanofiber and challenged with 5LD_50_ (1.75 × 10^4TCID50) of avian influenza H7N9 virus (A/Shanghai/4664T/2013). The survival curves were analyzed by Kaplan–Meier methods (*N* = 8 in each group). **(E)** Average weight of eight mice challenged with avian influenza H7N9 virus in each group are shown. **(F)** Viral RNA copies in the lung tissues of survivors in each group challenged with influenza H7N9 virus are shown.

We evaluated the protective efficacy of vaccination with M2e-Q11 nanoparticles against homologous influenza virus in mice. Mice were challenged with lethal dose of mouse-adapted influenza PR8 H1N1 virus after the prime-boost immunization. All the mice showed signs of influenza disease such as huddling, ruffled fur from 3-day post-infection. As shown in Figure 5A, seven of eight mice in Q11-immunized group died after influenza virus infection. The median survival time in this group is 8 days. In contrast, five of eight M2e-Q11 vaccinated mice survived from influenza PR8 challenge. The survival rate is significantly higher than mice immunized with Q11 nanofiber (*P* = 0.0222, Log-rank test). M2e-Q11 immunized mice also exhibited less body weight loss than mice immunized with Q11 nanofiber (Figure 5B, *P* = 0.0249, two-way ANOVA). As a positive control, six of eight mice immunized with aluminum-adjuvant M2e peptide survived from influenza challenge. However, neither survival rates or body weight change differ significantly between the mice immunized with aluminum-adjuvant M2e peptide and M2e-Q11 nanoparticles. The pulmonary viral titers in survivors of M2e-Q11 immunized group were lower than those of aluminum-adjuvant M2e peptide but the difference was not significant (Figure 5C, *p* = 0.4875, *t*-test).

We next evaluated whether the M2e-Q11 nanoparticle could provide cross-protection against heterologous influenza viruses. Mice were challenged with highly pathogenic avian H7N9 influenza virus after the prime-boost immunization. As shown in Figure 5D, five of eight mice in Q11 immunized group died from avian H7N9 influenza infection. The median survival time in this group was 12 days. However, all the mice immunized with M2e-Q11 or aluminum-adjuvant M2e peptide survived from avian H7N9 influenza infection. The survival rates in these two groups were significantly higher than Q11-immunized group (*P* = 0.0004, Log-rank test). The mice immunized with M2e-Q11 nanoparticles and aluminum-adjuvant M2e peptide also showed loss of body weight, but significantly less than mice immunized with Q11 nanofiber (Figure 5E, *P* < 0.0001, two-way ANOVA). However, no significant difference was observed in the pulmonary viral titers among the survivors of the three groups (Figure 5F, *P* = 0.6505, one-way ANOVA).

## Discussion

The application of nanotechnology is a promising strategy for development of effective vaccines against infectious viruses (Chen et al., 2013). Multiple nanotechnology platforms including polymeric nanoparticles, self-assembly proteins and peptides, inorganic gold nanoparticles have been investigated for the development of influenza vaccines (Al-Halifa et al., 2019). Here, we used the fibrillizing peptide Q11 to form an influenza M2e-based nanoparticle vaccine. The M2e-Q11 peptide successfully self-assemble into nanoparticles in physiological salt solution and induced antibody responses against different subtypes of influenza M2e peptides in experimental mice. This self-assembling M2e nanovaccine also protected mice against multiple subtypes of influenza viruses, include both group 1 (mouse-adapted H1N1 PR8) and group 2 (avian influenza H7N9) influenza viruses.

One advantage of using self-assembling peptide vaccine over protein-based peptide vaccines is the low immunogenicity of the carriers. Self-assembling peptides usually do not induce immune responses (Chen et al., 2013). In our study, neither M2e-Q11 nor Q11 nanoparticle induced detectable Q11-specific antibodies in mice after a prime-boost immunization. The low immunogenicity of carriers may avoid potential side effects or carrier-induced epitopic suppression in clinical use. Another advantage of peptide-based nanovaccines is that peptides can be synthesized in high purity by current solid-phase peptide synthesis method without downstream purification (Rudra et al., 2010). It does not only reduce the production cost, but also lower the risk of potential side effects caused by the contamination of bacterial endotoxin or mammalian cell components during protein expression.

In research reported in this paper, we used the self-assembling Q11 domain to promote the formation of nanoparticles because it has been successfully used to enhance the immunogenicity of several antigenic epitopes (Rudra et al., 2010, 2012a,b). Other self-assembling peptides may also have similar adjuvant activities. However, the ability of forming nanoparticles of self-assembling peptides needs to be evaluated when linked to antigenic epitopes. Q11 conjugates successfully self-assembled into nanofiber and induced strong humoral and T cell immune responses when incorporated with short antigenic epitopes such as ova, malaria and influenza T cell epitopes (Rudra et al., 2010, 2012a,b; Si et al., 2018). While M2e-Q11 conjugate in this study failed to form nanofibers, it assembled into short nanosticks instead. The M2e epitope consists of 23 amino acids, which is longer than ova, malaria, and influenza T cell epitopes (Mezhenskaya et al., 2019). The structure of M2e peptide may prevent Q11 from forming long nanofibers. In a previous report, a conjugate of Q11 peptide linked with a 29-mer B cell epitope J14 from streptococcus, failed to form nanostructure and did not induce specific immune responses (Azmi et al., 2014). These results indicated that the adjuvant activity of fibrilized peptide is dependent on its ability of forming nanoparticles. How the differences of nanoparticle size and structure influence the antigen presentation and immune responses is still under investigation.

Comparing with traditional inactivated or live-attenuated influenza vaccines, immune response induced by M2e-based vaccines were not able to prevent influenza infection (Heinen et al., 2002). Mice immunized with M2e-Q11 nanoparticles or aluminum-adjuvant M2e peptide still got infected and showed signs of disease such as huddling, ruffled fur and weight loss after challenge with influenza viruses. However, M2e-Q11 nanoparticle could provide cross-protection and significantly reduced the mortality of mice after infection with either group 1 or group 2 influenza subtypes. The protective mechanism(s) mediated by M2e-Q11 nanoparticles is still under investigation. Previous studies showed that M2e-sepcific antibodies contributed to protection induced by M2e-based vaccines (Deng et al., 2015; Rappazzo et al., 2016). However, M2e-Q11 nanoparticles induced comparable protection against PR8 virus as aluminum-adjuvant M2e peptide despite that it induced lower titers of anti-M2e antibodies than aluminum-adjuvant M2e peptide. Furthermore, although there were more than 25% (6/23) amino acid substitutions between the M2e antigen and influenza H7N9 M2e sequences, M2e-Q11 nanoparticle still provided complete protection against avian influenza H7N9 virus. These results indicated that M2e-Q11 nanoparticles induced immune responses against the conserved regions of M2e domain and so could be a promising candidate for universal influenza vaccine.

## Data Availability Statement

All datasets generated for this study are included in the article/supplementary material.

## Ethics Statement

The animal study was reviewed and approved by Institutional Animal Care and Use Committee of Shanghai Public Health Clinical Center.

## Author Contributions

FW, JH, QW, and YZ conceived and designed the experiments. QW, YZ, PZ, MW, WF, JS, and ZS performed the experiments. FW, JH, QW, YZ, and JX analyzed the data. FW, JH, QW, and YZ wrote the manuscript. All authors contributed to the article and approved the submitted version.

## Conflict of Interest

The authors declare that the research was conducted in the absence of any commercial or financial relationships that could be construed as a potential conflict of interest.
